# Fostering college students’ mental well-being: the impact of social networking site utilization on emotion management and regulation

**DOI:** 10.1186/s40359-024-02186-7

**Published:** 2024-11-21

**Authors:** Yuehua Han, Zhifen Xu

**Affiliations:** School of Business, Xinyang Normal University, Xinyang, Henan 464000 China

**Keywords:** Social networking site, Mental health, Emotion management, Multimodal data, Sentiment analysis

## Abstract

With the widespread proliferation of the Internet, social networking sites have increasingly become integrated into the daily lives of university students, leading to a growing reliance on these platforms. Several studies have suggested that this emotional dependence on social networking sites stems from unmet psychological needs. Meanwhile, social rejection has been identified as a prevalent phenomenon that exacerbates the deficiency of individual psychological needs. However, existing research on aspect-level sentiment analysis among college students within social networking sites faces challenges such as inadequate feature extraction, ineffective handling of data noise, and the neglect of complex interactions in multimodal data. To address these issues, this paper introduces a novel approach, the Multi-Granular View Dynamic Fusion Model (MVDFM), developed from both coarse-grained and fine-grained perspectives. MVDFM extracts multi-granular view features from textual and visual content, incorporating a dynamic gating self-attention mechanism. Additionally, it proposes a three-view decomposition higher-order pooling mechanism for a two-stage dynamic fusion of these features. Experimental results demonstrate the model’s effectiveness, achieving accuracy and F1 values of 78.78% and 74.48% on the Twitter-2015 dataset, and 73.89% and 72.47% on the Twitter-2017 dataset, respectively. This efficient supervision enables the extraction of deep semantic information from multimodal data generated by college students on social networking sites. The model adeptly mines pertinent information related to target aspect-based words, enhancing the efficacy of aspect-level emotion prediction. Furthermore, it facilitates an effective exploration of the intricate interplay between social rejection, monitoring on social networking sites, the fear of missing out, and dependence on social networking sites, ultimately aiding university students in regulating their emotional management.

## Introduction

While social networking sites function as technological mediators, and communication studies address technical concerns, the domain of psychology delves into the concealed psychological needs underlying this technological efficacy and the influencing mechanisms. The user base of social networking sites continues to burgeon annually, with site functionalities iterating to align with evolving user demands. Traditional instant messaging and basic personal information no longer suffice for contemporary users. The increasing sharing of personal information provides a more detailed picture of individuals, fostering a proclivity among users to seek more information, potentially leading to social networking site dependence.

Diverging from the common perception of “Internet addiction,” social networking dependence not only emphasizes usage duration but also underscores the interplay between an individual’s goal attainment abilities and their capacity to create, gather, process, and disseminate information on social networks [1]. Numerous studies have corroborated that social networking site addiction heightens levels of depression and anxiety, diminishes academic performance, and diminishes the overall psychological well-being of students [2]. Specifically tailored as an interactive platform for college students, social networking sites facilitate the expansion of social circles and the augmentation of opportunities for interpersonal engagement. When mitigating social networking site dependence, judicious use of these platforms can foster the psychological well-being of college students, aiding in stress alleviation, bolstering their sense of self-identity, and fortifying social support. Furthermore, the diverse content and abundant information on social networking sites cater to college students’ curiosity and thirst for knowledge. By perusing and assimilating such information, college students can enrich their knowledge base and elevate their cognitive proficiency, thereby enhancing their emotional management capabilities.

However, the utilization of social networking sites inevitably brings about some adverse consequences. Comments and visual content on these platforms can evoke potent emotional responses from college students. Excessive reliance on social networking sites may result in a distancing of real-life relationships and a decline in face-to-face communication skills. Additionally, negative information, such as unfounded criticism or attacks, occasionally surfaces on social networking sites, and prolonged exposure to such environments may impose stress on the mental health of college students, impacting their emotional management.

To foster the cultivation of resilient mental states among college students confronted with negative information on social networking sites, scholars in the research community have advocated for aspect-level sentiment analysis. This analytical approach aims to discern the sentiment tendencies of individual aspectual entities within textual expressions [3]. In contrast to conventional sentiment analysis tasks, this fine-grained sentiment analysis provides more nuanced and targeted insights, aligning closely with the intricacies of genuine emotional cognition, thereby garnering substantial attention from academia. However, prevailing sentiment classification methods predominantly rely on textual data, neglecting the valuable auxiliary information that multimodal data, such as images and videos, can furnish.

In recent years, propelled by advancements in multimedia technology, the content consumed by college students on social networking sites has embraced a multimodal trajectory. Beyond mere textual comments or tweets, users now incorporate data with diverse modalities, including images, to enrich their expressive viewpoints [4]. These interconnected multimodal data [5] possess untapped potential for intuitive expression and the conveyance of opinionated information. Consequently, integrating information from text-related images becomes imperative to fortify the robustness of aspect-level sentiment analysis models. It necessitates the dynamic emphasis of focal elements in each feature vector based on distinct target aspect words, thereby augmenting the quality of view feature generation. In response, this paper introduces the Multi-Granularity View Dynamic Fusion Model (MVDFM) with the ensuing key contributions:

1. From both coarse-grained and fine-grained perspectives, vectorization and coding of text and image data on social networks are executed to comprehensively capture data features and enhance the information representation of the model.
2. Extraction of multi-granularity view features from text and images is achieved, complemented by the design of a dynamic gating self-attention mechanism for noise reduction at the fine-granularity level, ensuring the meticulous quality of feature extraction.
3. To exploit the complementarity and coherence across multiple views at distinct granularities, a Triple-View Factorized High-order pooling (TFH) mechanism is introduced. This mechanism facilitates a two-stage dynamic fusion of multi-granularity view features, culminating in the derivation of the final sentiment polarity of the target aspect word.

Section 2 provides an overview of the current landscape in social networking site dependency and aspect-level sentiment analysis. The construction of the MVDFM is detailed in Sect. 3. Section 4 is dedicated to elucidating experimental results, scrutinizing the scheme’s performance, conducting a comparative analysis with classical schemes, and presenting ablation experiments that dissect the role of each module in the model. Furthermore, the section delves into exploring the interplay between social exclusion, social networking site monitoring, the fear of missing out, and social networking site dependency under affective prediction.

## Related works

### Social networking site dependency

On social networking sites, college students curate personal profiles that enables the linkage of their individual pages with those of friends. These friends, granted access to profiles, home pages, and other information, can engage in instantaneous messaging at their convenience. In certain communication studies, a proclivity exists to differentiate between social networking sites and social media. Nonetheless, within the realms of psychology and select communication research, there persists a belief that a distinct demarcation between the two is unnecessary. Unlike their early singular functionalities, contemporary popular domestic social networking sites now exhibit attributes akin to social media platforms. For instance, on WeChat, the circle of friends facilitates the sharing of web links, while QQ permits the perusal of copious information within a user’s space. Simultaneously, social networking sites have assimilated various social attributes. Platforms like microblogging enable reciprocal attention for viewing homepages and instant messaging. In addressing deleterious habits associated with social networking sites, the research community employs terminology such as social networking site addiction and problematic social networking site use. Notably, social networking site dependence is emerging as a focal point within this discourse.

Literature [6] has outlined internet dependence, segmenting it into five subtypes. Social networking site dependence, an expansive concept, has been elucidated through various perspectives. Some studies extend it from the foundation of media and technology dependence. Initially, scholars introduced the overarching concept of technology dependence into the realm of social networking site dependence. Certain experts posit that technology dependence serves as a pivotal determinant influencing behavioral habits in using social networking sites. Conversely, other studies build upon the framework of psychological trust, contending that dependence is fundamentally a psychological state. Dependence is further categorized into material dependence, primarily associated with substance abuse, and psychological dependence. Literature [7] defines social networking site dependence as an incapacity to regulate one’s usage of these platforms due to a deficiency in self-regulation within the context of psychological dependence.

Additionally, literature [8] posits that social media dependence revolves around an individual’s perception of the pivotal role social media plays in achieving daily life goals. It is also considered a manifestation of addictive behavior, emphasizing the psychological state associated with using social networking sites. Social media dependence is delineated as a dual manifestation, involving both psychological and behavioral dependence, stemming from a lack of self-regulation within the context of psychological dependence [9]. Concurrently, the utilization of social networking sites leaves an imprint on college students’ self-perception. The interactive platforms within social networks facilitate an understanding of peers’ life statuses and garner evaluations and feedback about oneself from others [10]. This interactive process enables college students to enhance their self-awareness through social comparison and attain opportunities for self-improvement. Nevertheless, it is acknowledged that this interaction may also wield a negative impact on college students’ self-perception.

Other research scholars have explored how news recommendation systems with values and diversity perception affect readers’ news choices [11], focusing on the impact of misinformation generated by generative AI on user information processing [12], and how people identify misinformation in health advice provided by AI [13]. These provide a rich background and theoretical support for the study of the effects of social networking site use on college students’ mental health and emotional management. They reveal the effects of algorithms, information recommendation and misinformation on user psychology and behavior, and provide guidance for formulating effective intervention strategies.

In summary, the impact of social networking site use on college students’ mental health and emotion management regulation is intricate. While it can, to a certain extent, mitigate loneliness, enhance the sense of social support, and augment emotion management skills, an excessive dependence on social networking sites may engender adverse effects. Consequently, college students are advised to adhere to the principle of moderation, enjoying the convenience and amusement offered by social networking sites while remaining vigilant about potential risks. In light of this, scholars in the research community advocate for the implementation of affective predictive analytics to proactively manage and regulate college students’ emotions on social networking sites, fostering a trajectory toward healthy psychological development.

### Sentiment analysis

Amidst the advancement of deep learning techniques, a plethora of studies has embraced the integration of deep neural networks to autonomously extract feature representation information pertaining to aspectual words for aspect-level sentiment analysis. Long Short-Term Memory (LSTM) [14], a venerable model in natural language processing, renowned for its robust sequence modeling capabilities and is frequently employed to tackle aspect-level sentiment analysis challenges. Literature [15] introduces the Target-Dependent Long Short-Term Memory (TDLSTM) model, an enhancement of LSTM. This model utilizes LSTM for feature extraction on aspectual words, their preceding, and succeeding texts, enabling the prediction of aspect-level sentiment. While LSTM demonstrates commendable performance, its limitation lies in extracting sequential features without the ability to selectively emphasize pertinent viewpoint information related to aspectual words. This lacuna is adeptly addressed by the attention mechanism [16], which calculates weights to gauge the significance of different words in the text. The attention mechanism has emerged as a ubiquitous model in aspect-level sentiment analysis.

Building upon LSTM networks to discern the latent semantics of context and aspectual words, literature [17] devises an Interactive Attention Network (IAN) rooted in the attention mechanism. IAN accurately predicts sentiment polarity by directing attention towards the words most germane to aspectual words. In a bid to fortify the interrelations between aspectual words and their corresponding viewpoint information, literature [18] introduces a novel model leveraging Graph Convolutional Network (GCN) [19]. GCN is utilized to model the syntactic dependency tree of a text, thereby mining dependencies between syntactic information and words. This approach aims to mitigate issues of misalignment between aspects and irrelevant viewpoint words.

While the aforementioned methods have significantly propelled the progress of aspectual sentiment analysis, they predominantly focus on the examination of textual modal data, neglecting the informative contributions of other multimodal sources such as pictures and videos. These visual elements, increasingly prevalent in user-generated content, hold significant reference value for discerning the sentiment polarity of aspectual words. Addressing this gap, literature [20] pioneers the domain of multimodal aspect-level sentiment analysis by introducing the Multi-Interactive Memory Network (MIEN). MIEN employs a multi-hopping attention mechanism to model the intricate interactions among aspect words, text, and images, predicting the sentiment polarity of predefined aspect categories. Subsequent research, as exemplified by literature [21], delves into the interplay between target aspect words and text and image data. Utilizing the attention mechanism, local features pertaining to aspect words in textual and visual representations are mined, and the fused features contribute to improved predictions. The literature [22] focuses on emotion-driven distress identification and cause extraction within multimodal online posts. On social networking sites, university students frequently express their emotions and difficulties through various forms such as text, images, and videos. The method proposed in this paper standardizes the analysis of these multimodal data, enabling more accurate recognition of students’ emotional distress and extraction of the causes underlying their difficulties. Meanwhile, literature [23] explores the application of computational intelligence in natural language processing and sentiment analysis. Sentiment analysis is a crucial technique for understanding the emotions and opinions expressed in text, which is essential for analyzing the speech and emotional states of university students on social networking sites. Additionally, literature [24] introduces a context- and knowledge-enriched Transformer framework for recognizing emotions in conversations. Conversations among university students on social networking sites often contain rich contextual information and emotional exchanges, and the method proposed in this paper allows for more precise identification of emotions within these conversations.

Building upon this foundation, researchers have endeavored to enhance model performance by elevating the quality of feature generation. Literature [25] devises the Entity-Sensitive Attention and Fusion Network (ESAFN), leveraging the attention mechanism to capture dynamic relationships between aspect words and text/image data. This approach aims to enhance the quality of visual features by mitigating noise in the image data. Literature [26] introduces the Hierarchical Interactive Multimodal Transformer model (HIMT), featuring an auxiliary reconstruction module to diminish semantic disparities between images and text, thereby improving multimodal feature fusion. Meanwhile, literature [27] proposes the Cross-Modal Multitask Transformer (CIMT), employing auxiliary task control models like named entity recognition and image aspectual emotion prediction to generate aspect-word-aware textual and visual representations. A cross-modal interaction module is introduced for the final feature fusion to predict the sentiment polarity of target aspect words.

Despite the diversity of approaches, common challenges persist, particularly in insufficient feature extraction and the neglect of noise in text and image modalities unrelated to target aspect words. Consequently, for the task of aspect word sentiment prediction, further research is warranted to expand feature extraction, ensuring comprehensive information representation, and to address noise reduction in relevant text and image data, thereby preserving the quality of feature representation.

## Methodology

**Fig. 1:**
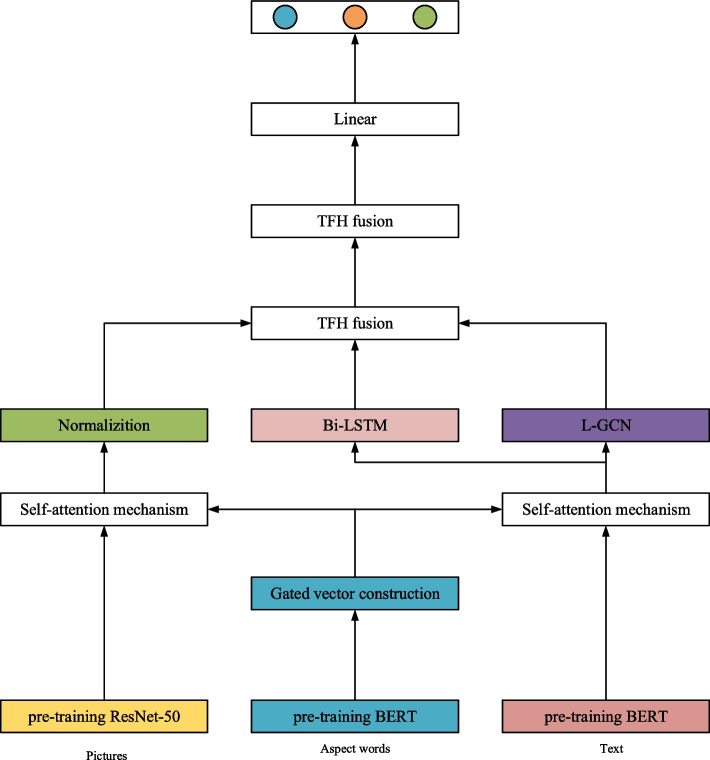
Model framework

The proposed method for multimodal aspect-level sentiment analysis, as delineated in this manuscript, encompasses key processes such as the encoding of multimodal data, dynamic extraction of multi-granular view features, fusion of multigranular view features, culminating in multimodal aspect-level sentiment prediction. The schematic representation of the model structure are illustrated in Fig. 1. During multimodal data encoding, we leverage the text pre-training model, BERT, and the image pre-training model, chosen to encode text, aspect words, and images at varying granularity levels. This preparation is crucial for generating subsequent multigranular view features.

The multi-modal aspect-level sentiment analysis method proposed in this paper, which is based on dynamic fusion of multi-grained views, primarily encompasses processes such as multi-modal data encoding, dynamic extraction of multi-grained view features, fusion of multi-grained view features, and ultimately, multi-modal aspect-level sentiment prediction. The specific model structure is illustrated in Fig. 1. During the multi-modal data encoding phase, pre-trained text model BERT and image pre-trained models are selected to encode text, aspect terms, and images at different granularity levels, respectively, laying a solid foundation for the subsequent generation of multi-grained view features. Notably, the dynamic extraction module of multi-grained view features incorporates the core component of the MVDFM architecture—the dynamic gated self-attention mechanism. This mechanism dynamically adjusts the importance of features at different granularity levels based on contextual information and task requirements, effectively capturing crucial information. Specifically, it utilizes gating units to weight coarse-grained and fine-grained features, enabling the model to adaptively focus on the most critical feature views for sentiment analysis.

Furthermore, the MVDFM architecture encompasses an innovative triple-view decomposition high-order pooling mechanism. This mechanism targets the fused multi-grained view features and processes global views, regional views, as well as sequential and syntactic views through three independent branches, achieving high-order abstraction and detailed decomposition of features. Each branch employs advanced high-order pooling techniques to deeply explore the complex relationships between features and extract more representative high-order features. These high-order features are then integrated, further enhancing the model’s expressive power and generalization ability. Lastly, during the dimensionality reduction of the fused features, the model ingeniously utilizes activation functions to map high-dimensional features into a low-dimensional space while preserving critical sentiment information. Through this process, the model generates a prediction probability vector that accurately reflects the distribution probabilities of aspect terms across different sentiment categories, thereby achieving precise classification of aspect-level sentiments.

### Multimodal data encoding

To fully harness the potential of multimodal data and comprehensively extract the rich information within, this study adopts a strategy of encoding images and text based on extraction granularity. For images, we leverage deep learning models pre-trained on large datasets to extract both global and regional features. Global features represent the overall image content, while regional features focus on specific parts of the image that are likely to contain sentiment-related information. To mitigate noise, we apply techniques such as data augmentation and regularization during model training, enhancing the model’s ability to generalize and filter out irrelevant information. Specifically, the text pre-training model, BERT, and the image pre-training model, ResNet50, are employed for the encoding of text, aspect words, and images, respectively. In the case of text and aspect words, a preprocessing step involves converting them to “[CLS] + text+[SEP]” and “[CLS] + aspect word+[SEP]” before inputting them into the BERT model for encoding. As for images, they undergo resizing to 224 × 224 pixels and are converted to the RGB three-channel format before being subjected to feature extraction using ResNet50.

Based on the above description, five information representations will eventually be derived. The text vectorization at sentence granularity output by the [CLS] tag of the BERT model is denoted as$H_{s}^{0} \in {{\mathbb{R}}^{1 \times 768}}$. The image vectorization at image granularity output by the last layer of ResNet50 is denoted as $H_{i}^{0} \in {{\mathbb{R}}^{1 \times 768}}$. The text vectorization at word granularity output by BERT is denoted as$H_{s}^{{}} \in {{\mathbb{R}}^{n^\prime \times 768}}$ and the aspectual word vectorization is denoted as ${H_a} \in {{\mathbb{R}}^{r^\prime \times 768}}$. The image vectorization at region granularity output by the penultimate layer of ResNet50 is denoted as ${H_i} \in {{\mathbb{R}}^{49 \times 768}}$ where $n^\prime$ and $r^\prime$ denote the encoded text and aspectual word sequence lengths,49 and the number of subregions into which an image is divided.

### Feature extraction

To extract global view features for both text and image, this paper amalgamates the vectorized representation of text at the granularity of sentence vectors and the vectorized representation of the overall image granularity. This fusion incorporates aspect word feature information, including contextual context information, for both text and image.

Firstly, the context subsequence $\left\{h_s^{k+1},h_s^{k+2},\dots,h_s^{k+r}\right\}$ is extracted as the aspect word feature matrix with contextual information based on the pre-recorded start position and the length of the aspect word, and secondly, it is subjected to average pooling operation to generate $H_{{a^\prime}}^{0}$. The feature vector generated by adding $H_{s}^{0}$ and $H_{{a^\prime}}^{0}$ vectors through the self-attention mechanism is used as the text global view feature $View_{s}^{{Glo}}$, and the feature vector generated by adding $H_{i}^{0}$ and $H_{a^\prime}^{0}$ through the self-attention mechanism is used as the image global view feature $View_{i}^{{Glo}}$, and the specific procedure is as follows.

1 $$H_{a^\prime}^{0}=\tfrac{1}{n}\sum\limits_{n=1}^{r} {h_{s}^{k+n}}$$

2 $$View_{s}^{{Glo}}=Attention\;(H_{a^\prime}^{0}+H_{s}^{0})$$

3 $$View_{i}^{{Glo}}=Attention\;(H_{{a^\prime}}^{0}+H_{i}^{0})$$

Where r refers to the aspect word text length.

**Fig. 2:**
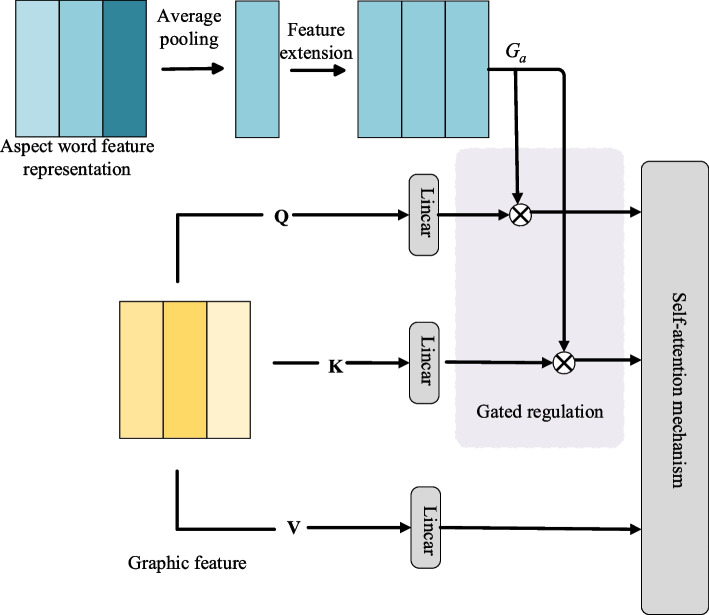
Dynamic gated self-attention mechanism

The fine-grained level view features that offer a richer expression of information, but concurrently, they tend to incorporate considerable noise unrelated to the sentiment expression itself. This redundant information is absolute noise. Furthermore, in instances where a single sample contains multiple aspect words with varying sentiment polarities, the information pertinent to aspect word A may be perceived as noise relative to aspect word B. To accurately extract viewpoint information related to the target aspect word, it is imperative to undertake noise reduction processing in the early stages of feature extraction to enhance the feature representation of both text and image. Accordingly, this paper introduces the dynamic gating self-attention mechanism, a module primarily grounded in the self-attention mechanism, enhanced with a specialized improvement in the gating mechanism, as illustrated in Fig. 2.

This module first utilizes the aspect word feature representation ${H_a}$ to construct the gating regulation vector ${G_a}$, and the specific process is shown in Eq. (4):

4 $${G_a}=\exp and(\sigma ({W_G} \times AvgPool({H_a})+{b_G}))$$

Where $\sigma ( \cdot )$ denotes the Sigmoid activation function, ${W_G}$ and ${b_G}$ are the trainable parameters, and $~expand( \cdot )$ refers to the lengthwise extended feature vector. Secondly, the gating adjustment vector ${G_a}$ is embedded into the multi-head self-attention mechanism by Hadamard dot-product, which participates in the generation of $H_{s}^{{{k_j}}}$ ,$H_{i}^{{{k_j}}}$, and $H_{s}^{{{Q_j}}}$ ,$H_{i}^{{{Q_j}}}$ in each attention head. The specific process is as in Eqs. (5) and (6).

5 $$\tilde {H}_{s}^{{{Q_j}}}=(1+G_{a}^{j}) \circ H_{s}^{{{Q_j}}},\tilde {H}_{s}^{{{k_j}}}=(1+G_{a}^{j}) \circ H_{s}^{{{k_j}}}$$

6 $$\tilde {H}_{i}^{{{Q_j}}}=(1+G_{a}^{j}) \circ H_{i}^{{{Q_j}}},\tilde {H}_{i}^{{{k_j}}}=(1+G_{a}^{j}) \circ H_{i}^{{{k_j}}}$$

Among them, $(1+G_{a}^{j})$ retains the features that have not been activated by the gating vector to enhance the activated features, and $\circ$ is the Hadammard dot product. The features $\tilde {H}_{s}^{{{k_j}}}$ ,$\tilde {H}_{i}^{{{k_j}}}$, and $\tilde {H}_{s}^{{{Q_j}}}$ ,$\tilde {H}_{i}^{{{Q_j}}}$ that have been adjusted by the gating vector will then be involved in the weighting process between Key and Query of the self-attention mechanism, at which time the calculated weights are dynamically adjusted. When assigning weights to the Value features, attention can be strategically directed towards pertinent text words or image regions. Specifically, higher weights are assigned to information associated with the target aspect words, while lower weights are allocated to information relatively unrelated to the target aspect words. This approach ensures that weights are tailored to different features based on their association with distinct aspect words. The information linked to the target aspect word is bestowed with a higher weight value, while information unrelated to the target aspect word is assigned a lower weight value. This dynamic weighting scheme facilitates effective noise reduction corresponding to different target aspect words. Finally, the text and image feature representations ${H_{sa}}$ and ${H_{ia}}$ which are sensitive to the aspect words are generated. The specific process is shown in Eqs. (7, 8, 9, 10).

7 $$H_{{sa}}^{j}=Attention(\tilde {H}_{s}^{{{Q_j}}},\tilde {H}_{s}^{{{k_j}}},\tilde {H}_{s}^{{{v_j}}})$$

8 $$H_{{ia}}^{j}=Attention(\tilde {H}_{i}^{{{Q_j}}},\tilde {H}_{i}^{{{k_j}}},\tilde {H}_{i}^{{{v_j}}})$$

9 $$H_{sa}=concat\left[H_{sa}^1;H_{sa}^2;\dots;H_{sa}^h\right]W_{sa}^h$$

10 $$H_{ia}=concat\left[H_{ia}^1;H_{ia}^2;\dots;H_{ia}^h\right]W_{ia}^h$$

Where $W_{{sa}}^{h}$ and $W_{{ia}}^{h}$ are trainable parameters and h denotes the number of heads of the multi-head attention mechanism.

The multi-granularity view feature fusion layer introduces a Triple-view Factorized High-order pooling (TFH) mechanism designed for this model. The TFH mechanism employs a two-phase form to fuse the upper-layer text global view features $View_{s}^{{Glo}}$, image global view features $View_{i}^{{Glo}}$, text order view features $Vie{w^{Seq}}$, text syntax view features $Vie{w^{Sny}}$, and image region view features to exploit the complementarity and consistency between different view features at different granularities and enhance the information representation capability. region view feature $Vie{w^{\operatorname{Re}g}}$ are fused to systematically investigate the complementarity and consistency across diverse view features at various granularities. This fusion process is aimed at enhancing the information representation capability of the merged features.

This paper initiates the design of TFB as the foundational stacking block for layer assembly to create the TFH, allowing for the extraction of deeper information. In contrast to coarse-grained view features, fine-grained view features encapsulate more nuanced local information. Consequently, these features are interactively fused, enabling the model to discern interactions among different local features in both images and text. To enhance the overall feature fusion process, this paper adopts a two-phase structure: first, achieving fusion interaction among fine-grained view features, followed by the fusion between coarse-grained views, culminating in the generation of the final fused features. The TFBs adeptly capture interactions between different features while preserving correlations among distinct modal features, serving as an effective fusion mechanism.

**Fig. 3:**
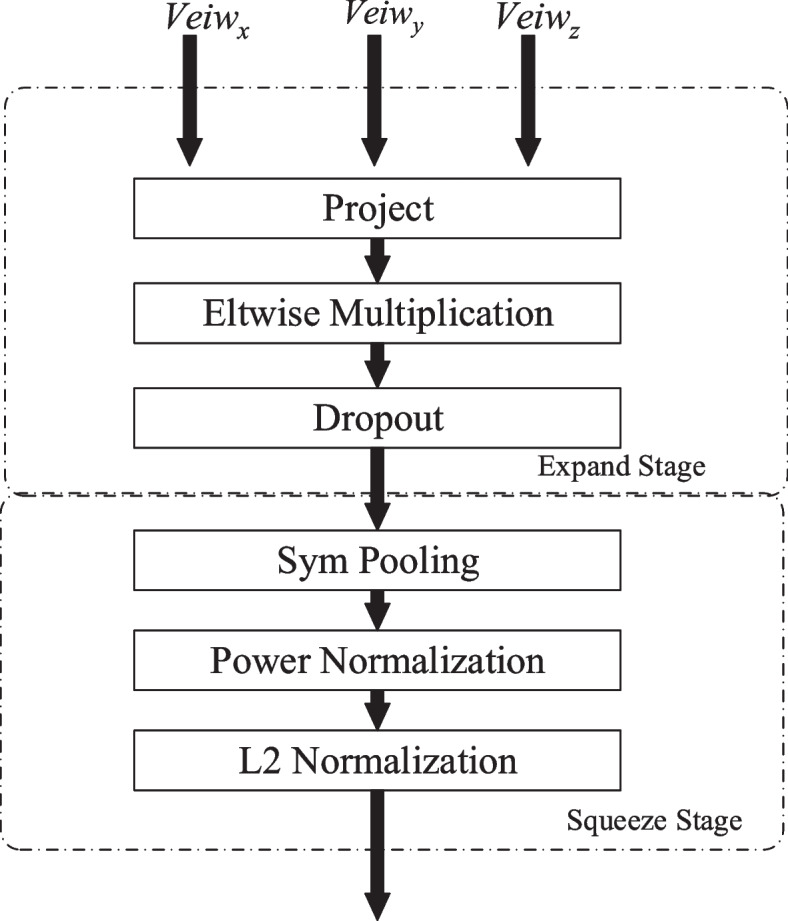
Three view decomposition bilinear pooling mechanism

Illustrated in Fig. 3, each TFB comprises two phases: feature expansion and feature compression. In the feature expansion phase, a projection layer is employed to project inputs into a consistent space and dimension. The Hadamard dot product operation integrates features, exploring their correlations, with a discarding layer to prevent overfitting. In the feature compression phase, the mechanism utilizes summation and pooling aggregation for the integrated information, incorporating Power normalization and L2 regularization to guard against local convergence in the model.

### Multimodal aspect-level sentiment prediction

The output layer aims to normalize the final fused feature output from the THF mechanism using a Softmax classifier, resulting in the probability values for each aspect word sentiment category. The sentiment associated with the maximum probability value is considered the model’s predicted result. The specific process is outlined as follows:

11 $$\hat {y}=soft\hbox{max} ({W_p}Vie{w^w}+{b_p})$$

Where $\hat {y}$ is the final sentiment prediction distribution, ${W_p}$ is the trainable parameters, and ${b_p}$ is the bias.

Acknowledging the ambiguity in defining neutral emotions and the potential unreliability of label values, this paper introduces Label Smoothing Regularization (LSR). LSR employs smoothed values instead of exclusively hot labels, providing the labels with a certain fault-tolerant space to enhance the model’s generalization performance. The generation process for smooth labels is articulated in Eq. (12).

12 $$y^\prime(k\vert x)=(1-\varepsilon)y(k\vert x)+\varepsilon u(k)$$

Where $y^\prime\left(k\vert x\right)$ denotes the newly generated smoothed label distribution of the sample x with label value k, $y(k|x)$ is the original label distribution of the sample x with label value k for the smoothing parameter value $\varepsilon$ is 0.2, $u(k)$ denotes the prior distribution of the label k In this paper, we set it to be $1/{\text{C}}$. The KL dispersion is used to measure it as shown in Eq. (13):

13 $${L_{lsr}}= - {D_{KL}}(u(k)||{p_\varsigma })$$

The final loss function consists of the cross-entropy loss and the ${L_{lsr}}$ ,${L_2}$ regularization terms as in Eq. (14):

14 $$L(\theta )= - \sum\limits_{{i=1}}^{C} {{{\hat {y}}^i}} \ln ({y^i})+{L_{lsr}}+\lambda \sum\limits_{{\theta \in \Theta }}^{{}} {{\theta ^2}}$$

Where in is the coefficient of the ${L_2}$ regularization term and C is the number of categories of the label.

## Experiments and analysis

This paper evaluates the model’s performance using two publicly available datasets: Twitter-2015 and Twitter-2017. Each dataset encompasses three sentiment polarities—positive, neutral, and negative—for every given aspect word. The inclusion of emotion-embedded data is valuable for assessing the model’s efficacy in regulating college students’ mental health and emotion management. The samples are partitioned into training, validation, and test sets in a 3:1:1 ratio. Model training is executed on the training set, with parameter adjustments based on experimental results from the validation set. The final performance evaluation is carried out on the test set.

The experiments were conducted using Windows 10 operating system, using PyCharmn2019.3 as the compilation software, based on the PyTorch deep learning framework, written in python3.8, and the models were trained and tested in an experimental environment of NVIDIAGeForceRTX3060 with 12GB of video memory.

### Evaluation indicators

To optimize the model performance, this study employed the Adam optimizer for parameter updates. Following several experiments, the optimal parameter combinations for the model on the Twitter-2015 and Twitter-2017 datasets were determined. The specific experimental parameter settings are detailed in Table 1.

**Table 1:** Experimental parameter settings

Hyperparameterization	Twitter-2015	Twitter-2017
Number of iterations	30	30
Batch size	32	32
Learning rate	1e-5	2e-5
Number of layer heads	8	8
Maximum encoding length of text	50	50
Maximum encoding length of aspect words	8	8

In this paper, the model’s effectiveness is assessed using accuracy and F1 value as evaluation metrics for the sentiment analysis task. Accuracy represents the overall performance of the model on all samples. The F1 value, a harmonized mean of accuracy and recall, serves to balance the two metrics. A higher F1 value indicates strong performance in both accuracy and recall. Notations include TP for true positives, TN for true negatives, FP for false positives, and FN for false negatives. The calculations for accuracy and F1 value are presented below:

15 $$Acc=\frac{{TP+TN}}{{TP+FP+FN+TN}}$$

16 $$\Pr e=\frac{{TP}}{{TP+FP}}$$

17 $$\operatorname{Re} c=\frac{{TP}}{{TP+FN}}$$

18 $$F1=\frac{{2*\Pr e*\operatorname{Re} c}}{{\Pr e+\operatorname{Re} c}}$$

### Model comparison under different datasets

**Fig. 4:**
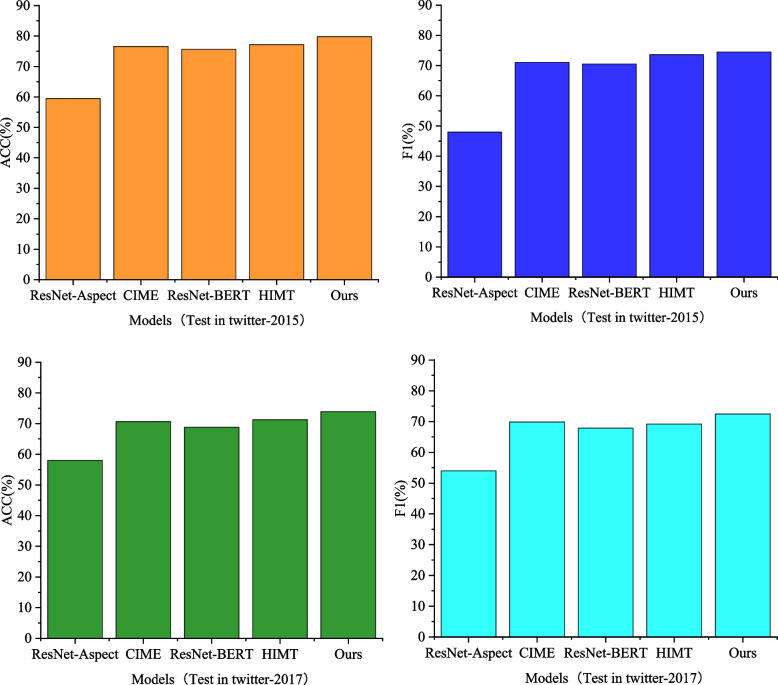
Comparison of model performance on different data sets

In the model comparison, the selected models for comparison include the image unimodal model ResNet-Aspect [28], the graphic multimodal model ResNet-BERT, CIME [27], and HIMT [16]. The conclusive experimental results are presented in Fig. 4.

In our model comparison, we selected the following baseline models: the image-only model ResNet-Aspect [21], and the multimodal image-text models ResNet-BERT [22], CIME [20], and HIMT [19]. The final experimental results, presented in Fig. 4, reveal that the image-only model ResNet-Aspect performed the worst among all models, achieving an accuracy and F1 score of 59.47% and 47.98% respectively on the Twitter2015 dataset. This suggests that the information related to aspect terms contained in images is limited, and text contains more valuable and exploitable content than images, serving as a crucial source of information for aspect-level sentiment analysis.

For text-only models, current research has shown that the pre-trained model BERT exhibits excellent representation capabilities for text content and can effectively learn context-related information, thereby enhancing model performance. Therefore, we also chose BERT to encode the text and aspect terms in our proposed model, MVDFM.

Furthermore, compared to BERT, ResNet-BERT achieved improved model performance, indicating that the incorporation of image data is beneficial for aspect-level sentiment analysis. Images enrich text information and can provide supplementary or supportive roles to text, thereby enhancing the effectiveness of sentiment classification.

Among all multimodal image-text models, CIME and HIMT significantly outperformed ResNet-BERT, which directly concatenates text and image features. This is because these multimodal models consider the matching relationship between aspect terms and multimodal data, and can reasonably design network architectures tailored to the characteristics of multimodal data to model the interaction between text and images, thereby improving the accuracy of aspect-level sentiment prediction.

Finally, our proposed model, MVDFM, achieved the best experimental results on both the Twitter-2015 and Twitter2017 datasets. Compared to the current state-of-the-art model HIMT, MVDFM improved accuracy by 0.55% and 1.67%, and F1 score by 0.88% and 2.45% respectively. On the Twitter2017 dataset, MVDFM achieved an accuracy and F1 score of 73.89% and 72.47% respectively. These results demonstrate that the overall design of the MVDFM model has significant advantages compared to other multimodal models. It can effectively utilize information from relevant text or images to predict sentiment polarity for different aspect terms, achieving good prediction performance.

Importantly, while the experiments primarily evaluated the performance of the models, future research should explore the practical applications of these models in mental health or emotion management. Empirical studies or discussions directly targeting these areas would further enhance the impact of our research. For example, by analyzing frequently occurring sad or negative graphic-text content, potential mental health risks can be promptly identified, enabling early interventions.

### Ablation experiments

**Fig. 5:**
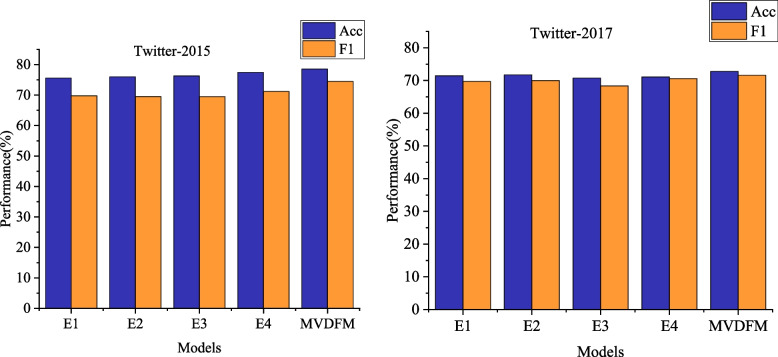
Results of ablation experiment

To further investigate the effectiveness of each component in the MVDFM model and its impact on overall performance, four sets of ablation experiments are designed. The experiments utilize the same two datasets, Twitter-2015 and Twitter-2017, and maintain consistent parameter settings with the full model. The specific ablation models are outlined as follows:

- E1: Remove the dynamic self-attention mechanism from the original model and directly employ feature splicing to fuse aspect word information with images and text.
- E2: Eliminate the text global view features and image global view features from the original model, retaining only the image region view features, text sequence view features, and text syntax view features for aspect-level sentiment prediction.
- E3: Exclude the text-image region view features, text-phrase order view features, and text-syntax view features from the original model, preserving only the global view features and the image global view features for aspect-level sentiment prediction.
- E4: Omit the TFH fusion mechanism from the original model and directly fuse the five view features using average pooling and feature splicing.

Figure 5 illustrates that the full model outperforms the ablated models in terms of performance. First, the removal of either coarse-grained or fine-grained level view features leads to a significant decrease in model performance, particularly evident in the Twitter-2015 dataset. The accuracy decrease can be around 3%. Analyzing data from different levels and perspectives allows for the expression of diverse features, providing varied information. The combination of these features facilitates mutual complementation and enhancement, enabling a more comprehensive extraction of effective information. This, in turn, expands the amount of information provided by the data, addressing challenges related to short text and insufficient data in the dataset.

Second, the complete model exhibits an accuracy improvement of 2.41% and 2.02% on the two Twitter datasets compared to the model without the dynamic gating self-attention mechanism. This suggests that the inclusion of the gating mechanism dynamically adjusts the computation of attentional weights, prioritizing information related to aspectual words. Consequently, the gating mechanism addresses redundant information and noise in the data.

Finally, the removal of the TFH mechanism has a noticeable impact on the model performance compared to the complete model. On the two Twitter datasets, accuracy is enhanced by 1.26% and 1.70%, respectively. This indicates that the TFH mechanism effectively taps into complex interactions among different features, ensuring comprehensive integration and further improving sentiment classification effectiveness.

In summary, MVDFM exhibits inherent flexibility by extracting multi-grained view features from text and images through both coarse-grained and fine-grained perspectives. When dealing with large datasets, the model can adjust the granularity of feature extraction according to needs, thereby optimizing the use of computational resources while maintaining performance. Secondly, the design of the dynamic gated self-attention mechanism enables the model to dynamically focus on key information in the input data, reducing the processing of non-key information and thus improving computational efficiency. This mechanism is particularly important when handling real-time sentiment analysis tasks, as it allows the model to quickly respond and process new data inputs. Furthermore, the triple-view decomposition high-order pooling mechanism proposed by MVDFM further enhances the model’s computational efficiency through two-stage dynamic fusion of multi-grained view features. This mechanism not only helps capture complex relationships in the input data but also reduces computational load through decomposition and pooling operations, thereby alleviating potential computational bottlenecks.

### Analysis of college students’ mental health and emotion management

To comprehensively and accurately capture the emotional features of text and image data on social networking platforms, we adopt a combined coarse-grained and fine-grained perspective for vectorized encoding. Coarse-grained analysis focuses on grasping overall emotional trends, such as the general distribution of positive, negative, or neutral sentiments. In contrast, fine-grained analysis delves into the details of text and image elements, identifying subtle cues like specific emotional vocabulary, emojis, and color schemes. This dual strategy significantly enhances the model’s information expression capabilities, laying a solid foundation for subsequent emotional analysis. Furthermore, we extract multi-grained view features from text and images and innovatively design a dynamic gated self-attention mechanism. This mechanism effectively filters out noise information, such as irrelevant vocabulary or blurred image areas, at the fine-grained level, ensuring high-quality and accurate feature extraction. This step is crucial for precisely identifying the complex emotions expressed by university students in graphic-text comments, providing a reliable basis for subsequent mental health assessments. To deeply explore the complementarity and consistency among different granularity views, we propose a view decomposition high-order pooling mechanism. This mechanism integrates multi-grained view features through a two-stage dynamic fusion strategy, ultimately outputting the emotional polarity of target aspect terms. This method not only improves the performance of graphic-text emotion prediction but, more importantly, it accurately analyzes the emotional information contained in the graphic-text content posted by university students on social networking sites, predicting their emotional states and indirectly reflecting their mental health conditions. For example, by analyzing frequently appearing sad or negative graphic-text content, potential mental health risks can be promptly identified, enabling early interventions.

**Fig. 6:**
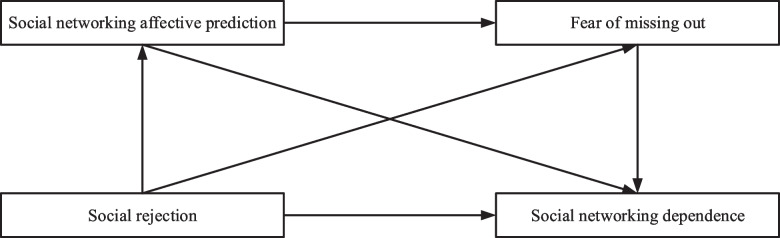
Emotional network analysis under affective prediction in social networking sites

Enhancing the performance of graphic emotion prediction allows for effective analysis of emotional information embedded in the text and images of college students when they make graphic comments on social networking sites. This predictive capability aids in understanding and detecting their mental health status. Specifically:

Detecting Psychological Distress: Text and image content on social networking sites serve as indicators of the emotional state of college students. Frequent posting of sad or negative texts and images over time may signal psychological stress or emotional problems. Emotion prediction can help identify potential issues and enable timely intervention and support.

Self-Regulation of Emotions: Graphic emotion prediction assists college students in understanding and regulating their emotions. Analyzing their text and image content allows students to recognize mood changes and emotional states. This self-awareness facilitates better emotional management, including the use of positive psychological suggestions, emotional catharsis, or seeking professional psychological counseling.

During the experimental exploration presented in Fig. 6, it was observed that social networking site dependence in college students falls into different qualitative potential categories and is influenced by factors such as social rejection, social networking site monitoring, and the fear of missing out. Additionally, social rejection is positively related to social website monitoring and fear of missing out. Social networking site monitoring mediates between social exclusion and social networking site dependence, while misplaced fear mediates between social exclusion and social networking site dependence. Furthermore, a chain mediation effect is observed, with social networking site monitoring and misplaced fear acting as mediators between social exclusion and social networking site dependence.

Interactions on social networking sites play a crucial role in enabling college students to form communities of support and mutual aid. Through communication and sharing with others who share similar experiences or feelings, students can garner emotional support and empathy, contributing to their mental health and emotional regulation. However, it is essential to recognize that graphic emotion prediction is not flawless. Factors such as individual language expression ability and cultural background can impact the accuracy of prediction results. Therefore, when employing this technique, it is crucial to consider various factors and integrate it with other mental health assessment methods to ensure accuracy and reliability.

### Discussion

In the field of law, particularly in litigation, contract review, legal research, and other areas, a vast amount of textual and image data requires analysis. Vectorizing and encoding text and image data from social networks through both coarse-grained and fine-grained perspectives can significantly enhance the digital processing capabilities of legal texts. This not only facilitates rapid retrieval and classification of legal documents but also enables automatic extraction of key information through machine learning algorithms, such as critical obligations in contract clauses and authenticity assessments of evidentiary materials, thereby enhancing the efficiency and accuracy of legal practice. Furthermore, this paper extracts multi-grained view features from text and images and designs a dynamic gated self-attention mechanism, which means that in the legal field, it becomes possible to more precisely identify and analyze key information in legal documents. The proposed Triple-view Factorized High-order Pooling (TFH) mechanism holds broad application prospects in the process of legal decision-making. By fusing multi-view information across different granularities, the model can more comprehensively understand the complexity of legal issues and provide more accurate legal advice. Combining these innovations, the prospects for Legal Master (LL.M.) graduates become broader and more diversified. On the one hand, with the continuous development of artificial intelligence and big data technologies, LL.M. graduates will have the opportunity to master more advanced digital tools and methods, enhancing their competitiveness and professional expertise in legal practice. On the other hand, LL.M. graduates can also leverage these technologies to conduct interdisciplinary research, such as in the cross-fields of law and computer science, law and data science, contributing to the development and innovation of the legal industry.

Moreover, with the advent of globalization and the Internet era, cross-border legal affairs are increasing, placing higher demands on legal talents with international perspectives and cross-cultural communication abilities. Therefore, during their training, LL.M. students should focus on international education and the accumulation of practical experience to adapt to the demands of the global legal market.

## Conclusion

This paper introduces a dynamic fusion model for multimodal aspect-level sentiment analysis, particularly focusing on graphic information from social networking sites. The model considers multimodal data from coarse-grained and fine-grained perspectives, extending various text and image view features. This expansion enhances the information content in multimodal data, ensuring more robust feature extraction for improved sentiment prediction, even in scenarios with limited multimodal data samples. To filter out noise unrelated to the target aspect word, a dynamic gating self-attention mechanism is proposed. This mechanism dynamically adjusts weight assignments for each word or region based on target aspect word information, effectively reducing noise in the data. Furthermore, a three-view decomposition higher-order pooling mechanism is employed for final feature fusion. This mechanism captures complex interactions among features from different viewpoints, achieving effective fusion through multiple expansion and compression stages. Experimental results demonstrate that the proposed model outperforms current baseline methods on the Twitter-2015 and Twitter-2017 datasets. The analysis indicates a strong correlation between graphical sentiment prediction and college students’ mental health and emotion management regulation on social networking sites. The technique presented in this paper not only helps identify potential psychological problems in college students, providing timely intervention and support, but also assists students in better understanding and regulating their emotions, thereby promoting their mental health and emotion management skills.

## Data Availability

No datasets were generated or analysed during the current study.
